# Regulation of Nutritional Metabolism in Transition Dairy Goats: Energy Balance, Liver Activity, and Insulin Resistance in Response to Berberine Supplementation

**DOI:** 10.3390/ani11082236

**Published:** 2021-07-29

**Authors:** Navid Ghavipanje, Mohammad Hasan Fathi Nasri, Seyyed Homayoun Farhangfar, Seyyed Ehsan Ghiasi, Einar Vargas-Bello-Pérez

**Affiliations:** 1 Department of Animal Science, Faculty of Agriculture, University of Birjand, Birjand 97175-331, Iran; hfathi@birjand.ac.ir (M.H.F.N.); hfarhangfar@birjand.ac.ir (S.H.F.); s.e.ghiasi@birjand.ac.ir (S.E.G.); 2 Department of Veterinary and Animal Sciences, Faculty of Health and Medical Sciences, University of Copenhagen, Grønnegårdsvej 3, DK-1870 Frederiksberg C, Denmark

**Keywords:** berberine, energy balance, insulin resistance, liver activity, transition period, Saanen goats

## Abstract

**Simple Summary:**

The transition period is largely marked by a decline in dry matter intake (DMI) that often leads to a negative energy balance (NEB) which, along with exaggerated insulin resistance (IR), increasing the mobilization of body fat reserves, leads to higher blood non-esterified fatty acid (NEFA) and/or β-hydroxybutyric acid (BHBA) concentrations. These confounding factors adversely affect animal health and lactation performance. This study evaluated the impact of pre- and post-partum berberine (BBR) supplementation as a novel approach to the regulation of nutritional metabolism in transition dairy goats. In summary, BBR supplementation (2 and 4 g/d) elevated the DMI and energy balance (EB) in pre- and post-partum goats, as well as enhancing liver activity indices, showing the potential of the new therapeutic strategy in the prevention of metabolic dysfunction in transition dairy goats and in attaining an improved lactation performance as well as health.

**Abstract:**

The objectives of this study were to evaluate the alleviating effects of the isoquinoline alkaloid berberine (BBR) on the energy balance (EB), glucose and insulin metabolism, and liver functionality in transition dairy goats, as reflected by blood metabolites and enzymes. Twenty-four primiparous Saanen goats were randomly allocated to four groups. Goats in each group received, ad libitum, the same basal diet during the pre- and post-partum periods of evaluation. Goats received daily0, 1, 2, or 4 g BBR (coded as CON, BBR1, BBR2, and BBR4, respectively). Dry matter intake (DMI) and milk yield were recorded daily. Blood samples were collected on days −21, −14, −7, 0, 7, 14, and 21 relative to kidding, and individual body condition scores (BCSs) were also recorded. Supplementation with either BBR2 or BBR4 increased (*p* < 0.05) pre- and post-partum DMI, increasing (*p* < 0.05) the intakes of net energy for lactating and metabolizable proteins. BBR2 and BBR4 increased (*p* < 0.05) post-partum milk production as well as fat-corrected milk (FCM), energy-corrected milk (ECM), and feed efficiency, indicating the alleviating effect of BBR on the negative energy balance (NEB) in transition goats. The daily ingestion of either 2 or 4 g BBR reduced (*p* < 0.05) plasma aspartate aminotransferase (AST), alanine aminotransferase (ALT), and alkaline phosphatase (ALP) and increased (*p* < 0.05) the dynamic change in the liver activity index (LAI) and liver functionality index (LFI), implying its hepatoprotective effect on transition goats. Overall, the results suggest that BBR supplementation of at least 2 g/d may help to ameliorate insulin resistance (IR) and fat metabolism disorders initiated by the NEB in transition dairy goats.

## 1. Introduction

Pregnancy, parturition, and the onset of lactation represent an enormous physiological challenge to the homeostasis of dairy animals, being a risk for their health and reproductive performance [1,2]. The transition period is largely marked by a decline in dry matter intake (DMI) that often leads to a negative energy balance (NEB) on account of the tremendous physiological changes and other confounding factors [1,3]. NEB leads to extensive mobilization of fatty acids from the adipose tissue, whereby amounts of non-esterified fatty acids (NEFA) are released into the blood circulation [4]. One portion of these NEFA is then transported into the mammary gland for milk fat synthesis, and the rest are utilized by the liver for energy generation via oxidation which results in higher blood NEFA and/or β-hydroxybutyric acid (BHBA) concentrations [5,6]. An elevated NEFA concentration is a typical hallmark of transition doe, and the insufficient metabolic capability of hepatic NEFA has been demonstrated to adversely affect animal health and to impair lactation performance as well as subsequent reproductive performance [7,8].

Insulin resistance (IR) develops during the periparturient period and leads to increases in the availability of glucose, amino acids, and fatty acids (FAs) to the mammary gland [9]. However, exaggerated IR during late pregnancy and early lactation could promote greater mobilization of body fat reserves and expose transition goats to metabolic dysfunctions [10]. High concentrations of blood NEFA also contribute to increased oxidative stress and a dysfunctional inflammatory response during the post-partum period [7]. Both increased circulating fatty acid concentrations and chronic, low-grade inflammation have been linked to IR in humans and laboratory animals [11,12]. Insulin resistance decreases the sensitivity of tissue to the presence of insulin and plays a major role in the development of many metabolic health disorders in humans, including obesity, type 2 diabetes, hypertension, and cardiovascular disease [12]. Furthermore, the increased levels of circulating NEFA and BHBA have been reported to induce immunosuppression and liver dysfunction, characterized by the induction of acute-phase proteins and an impairment in liver activity [13,14,15]. Therefore, dietary management strategies in the transition state should be considered to ameliorate IR and liver activity to optimize glucose and lipid metabolism.

Berberine (BBR), a natural yellowish isoquinoline alkaloid, is found in certain species of flowering plants such as *Berberidaccae*, *Coptis rhizomes,* and *Hydrastis Canadensis*. Traditionally, BBR was used in Iranian and Chinese medicine for the treatment of a variety of diseases including gastrointestinal infections, fevers, typhus, and diarrhea [16,17]. BBR can be manufactured as chloride or sulfate salts and is generally used for clinical purposes. Clinical research and animal studies have provided significant results showing that BBR can regulate glucose and lipid metabolism and attenuate insulin resistance [18].

The beneficial effects of BBR on increasing blood insulin and improving insulin sensitivity were first reported by Chen and Xie [19], and the mechanisms of action of this alkaloid have been evaluated in recent decades [20]. BBR is characterized as an AMP-activated protein kinase (AMPK) activator. AMPK is a key energy-sensing/signaling system in adipocytes and acts as a fuel gauge by monitoring cellular energy levels, e.g., the AMP/ATP ratio [21]. Activation of AMPK is well known to increase insulin sensitivity and regulate the mitochondrial function [22]. AMPK, as an energy-sensitive protein kinase, maintains glucose homeostasis and plays an important role in regulating tissue insulin sensitivity. Ko et al. [23] reported that BBR could act as an effective insulin-sensitizing and insulinotropic agent in cell culture (allosaurus cell line MIN6) studies. BBR also improves glucose metabolism through an insulin-independent pathway [24]. BBR may also favor insulin secretion, as shown both in vitro, in a model of an insulin-secreting cell line cultured in the presence of glucose, and in vivo, in BALB/c mice [25]. This study hypothesized that BBR supplementation during the peri-partum period ameliorates the negative effects of IR and metabolic challenges in dairy goats. As such, supplemental BBR would have a positive effect on the energy balance, liver function, and animal performance. Therefore, the objective of this study was to evaluate the alleviating effects of BBR on the energy balance, glucose and insulin metabolism, and liver functionality in transition dairy goats, as reflected by circulating blood metabolites and enzymes.

## 2. Materials and Methods

### 2.1. Ethics Statement

The animal feeding experiment was conducted at the University of Birjand, Faculty of Agriculture Research Station, located in Birjand, Iran, from August 2019 to December 2019. The animals used were cared for in accordance with the guidelines of the Iranian Council of Animal Care [26] (no. 19293).

### 2.2. Design, Animal Management, and Treatments

Twenty-four healthy primiparous Saanen dairy goats (BW = 42 ± 3.5 kg; BCS = 3 ± 0.5, mean ± SD) were fed a basal diet without or with 1, 2, and 4 g/d BBR, starting at day −21 relative to the expected parturition through 3 weeks post-partum in a completely randomized design. These pharmacologically utilized doses are the corresponding doses which have been used by other researchers on insulin resistance and glucose metabolism in nonruminant species [27,28,29] and humans [18,30]. Thus, experimental treatments included: 1—basal diet without BBR supplementation (CON); 2—basal diet + 1 g BBR/d; 3—basal diet + 2 g BBR/d; and 4—basal diet + 4 g BBR/d, which approximately correspond to 0, 25, 50, and 100 mg dietary BBR/kg BW, respectively. BBR was purchased from Bulk Supplement Factory (Bulk Supplements, Eastgate, Henderson, NV, USA), and based on the catalog, this product is Pure Berberine HCL powder and had no other ingredients. To assure BBR consumption, it was encapsulated (gelatin capsule, irancapsul, Tehran, Iran) in doses corresponding to the experimental treatments and orally administrated to each goat before morning feeding; the control group received empty ones. Gelatin capsules were not rumen-inert.

Two different diets consisting of 45% forage and 55% concentrate on a DM basis were formulated to be isocaloric (2.60 and 2.90 Mcal ME in per- and post-partum diets, respectively) and isonitrogenous (18.5% and 15.5% CP in per- and post-partum diets, respectively) and to meet NRC [31] requirements for the pregnancy and lactation periods. The ingredients and nutrient composition of the pre-partum (offered 50 days pre-partum through kidding) and post-partum (offered from day 1 to day 21 of lactation) diets are presented in Table 1. All goats were housed in individual pens (1.8 × 1.6 m), and considering the potential impact of diet change on the ruminal environment, goats received the same diet (pre-partum ration) from the far-off to the close-up period [32]. Diets were mixed daily and offered ad libitum (allowing 5–10% orts) as a TMR twice per day (06:00 and 16:00 h), with free access to water.

### 2.3. Data Collection, Calculations, and Analyses

#### 2.3.1. Dry Matter Intake, Body Weight, and Body Condition Score

The quantity of the supplied TMR and orts was weighed daily, and dry matter intake (DMI) was then calculated by subtracting the quantity fed minus refusals multiplied by the DM content of the TMR. Body weight (BW) was recorded on days −21, −14, and −7 relative to kidding, and on days 7, 14, and 21 post-partum. Body condition score (BCS) was taken by the same individual at days −21, −14, −7, 0, 7, 14, and 21 relative to parturition according to a 1-to-5 scale with quarter points, where 1 = emaciated, and 5 = obese, as described by Villaquiran et al. [33].

#### 2.3.2. Energy Balance

The calculation of the energy balance (EB; Mcal/d) for each doe was conducted according to NRC [31]. Pre-partum EB (EB_pre_) was calculated as follows:

EB_pre_ = NE_I_ − (NE_M_ + NE_P_)

where NE_I_ (Mcal/d) represents the intake of net energy and was estimated by multiplying the daily DMI by the calculated NE value of the diet (Table 1); NE_M_(Mcal/d) represents the maintenance requirement of NE and was defined as BW^0.75^ × 0.08;and NE_p_(Mcal/d) represents the NE requirement for pregnancy and was calculated using a given formula [31].

NE_p_ = (0.00318 × Days of gestation − 0.0352) × (kid birth weight/0.45)/0.218

Post-partum EB (EB_post_) was estimated using the following equation:

EB_post_ = NE_I_ − (NE_M_ + NE_L_)

where NE_L_ (Mcal/d) represents the lactation requirement of net energy and was calculated as: (0.0929 × Fat% + 0.0547 × Protein% + 0.0395 × Lactose%) × Milk yield (kg/d) [31].

In addition, metabolizable protein intake (MP_I_) was obtained from the following formula [31]: MP_I_ (g/d) = DMI (kg) × MP (g/kg DMI).

#### 2.3.3. Milk Yield and Milk Components

Goats were milked at 05:30 and 15:30 daily, and production was recorded at each milking. Milk samples were collected in plastic bottles containing potassium dichromate, a preservative, on days 0, 7, 14, and 21 post-partum from 2 consecutive milking sessions. Milk samples were shipped immediately to a laboratory and analyzed for fat, protein, lactose, total solids (TS), and non-fat solids (SNF) on an infrared milk analyzer (Milko Scan FT 200, Foss Electric, Hillerod, Denmark) according to the manufacturer’s instructions.

The fat-corrected milk (FCM) was calculated as: FCM (4% fat) (kg/d) = (milk yield, kg/d × 0.4) + (fat, kg/d × 15) [34].According to Reist et al. [35], the following formula was used to calculate the energy-corrected milk (ECM): ECM (kg) = (0.038 × g of crude fat + 0.024 × g of CP + 0.017 × g of lactose) × kg of milk/3.14.

#### 2.3.4. Biomarkers of Energy Balance and IR

Preprandial blood samples were collected from the jugular vein of each doe on days −21, −14, −7, 0, 7, 14, and 21 relative to parturition. The blood sample was collected into an evacuated heparinized tube, and plasma was separated, followed by centrifugation at 3000× *g* for 15 min; it was then stored (−80 °C) until analysis.

Plasma glucose was measured by an autoanalyzer (BT 1500, Biotecnica SpA, Rome, Italy) using commercial kits (Pars Azmoon Co., Ltd., Tehran, Iran) according to the manufacturer’s instructions. BHBA and NEFA were determined by commercial colorimetric kits (Randox Laboratories Ltd., Ardmore, UK) using the same autoanalyzer. The serum insulin level was measured using an enzyme-linked immunosorbent assay kit (Monobind Inc., Lake Forest, CA, USA). Intra- and inter-assay coefficients of variation for measuring insulin were 6.9 and 8.2%, respectively.

The revised quantitative insulin sensitivity check index (RQUICKI) and RQUICKI including BHBA, relative insulin sensitivity measures, were estimated as RQUICKI = 1/[log (glucose) + log (insulin) + log(NEFA)], and RQUICKI_BHB_ = 1/[log glucose + log insulin + log NEFA + log BHB] according to Abuelo et al. [36].

#### 2.3.5. Biomarkers and Quantification of Liver Function

Plasma concentrations of albumin (ALB), alanine aminotransferase (ALT), aspartate aminotransferase (AST), and alkaline phosphatase (ALP) were measured using commercial kits (Pars Azmun Co. Ltd., Tehran, Iran) by an autoanalyzer (BT 1500, Biotecnica SpA, Rome, Italy).

Two quantitative indices of liver function including the liver activity index (LAI) [3] and liver functionality index (LFI) [37] were modified to integrally evaluate the dynamics of liver function, as described by Sun et al. [38]. Briefly, the modified LAI calculation was carried out in 2 steps. The first considered the vitamin A, total cholesterol, and albumin of the transition dairy goats at days −21, −14,−7, 0, 7, 14, and 21, and the partial index of each parameter was calculated (albumin is presented as an example):

Partial index of albumin = (Plasma level of albumin at −7 d − Average of the albumin at 21 d in tested does)/SD of albumin at 21 d in tested does.

In the second step, the final modified LAI score of each goat was presented as follows: LAI = (partial value of albumin + partial value of cholesterol + partial value of vitamin A)/3. Likewise, the LFI for all goats was calculated in two steps based on plasma concentrations of albumin, cholesterol, and bilirubin. The subindex of each parameter was calculated using the following equation (V*i* represents the value at day *i* during the transition period):

Albumin subindex (Alb_sub_) = 50%V*_i_* + 50%(V_21_ − V*_i_*)

Cholesterol subindex (TC_sub_) = 50%V*_i_* + 50%(V_21_ − V*_i_*)

Bilirubin subindex (BIL_sub_) = 67%V*_i_* + 33%(V*_i_* − V_21_)

Afterward, the LFI was calculated as follows: LFI= (Alb_sub_ − 17.71)/1.08 + (TC_sub_ − 2.57)/0.43 − (TBIL_sub_ − 6.08)/2.17 (see Sun et al. [38] and Trevisi et al. [37] for a thorough explanation).

### 2.4. Statistical Analysis

A completely randomized design with 4 treatments (different BBR levels) and 6 replicates (goats) was used for the study. All data were statistically analyzed using the MIXED model procedure of SAS (version 9.2, SAS Institute Inc., Cary, NC, USA). The model was: y*ijkl* = μ + T*i* + A*j(i)* + W*k* + T*i* × W*k* + e*ijkl*, where y*ijkl* is the dependent variable, μ is the overall mean, T*i* is the fixed effect of treatments, A*j(i)* is the random effect of *j*th goat within *i*th diet, W*k* is the fixed effect of repeated measurements, T*i*× W*k* is the interaction fixed effect between T*i* and W*k*, and e*ijkl* is the residual error. The covariance structure with the best fit was unstructured (UN). Least square means were computed and tested for differences by Tukey’s test. For all data, significance was declared at *p* ≤ 0.05, and tendencies were identified when the *p*-value was >0.05 and ≤0.10.

## 3. Results

### 3.1. Dry Matter Intake, Body Condition Score, and Energy Balance

**Figure 1:**
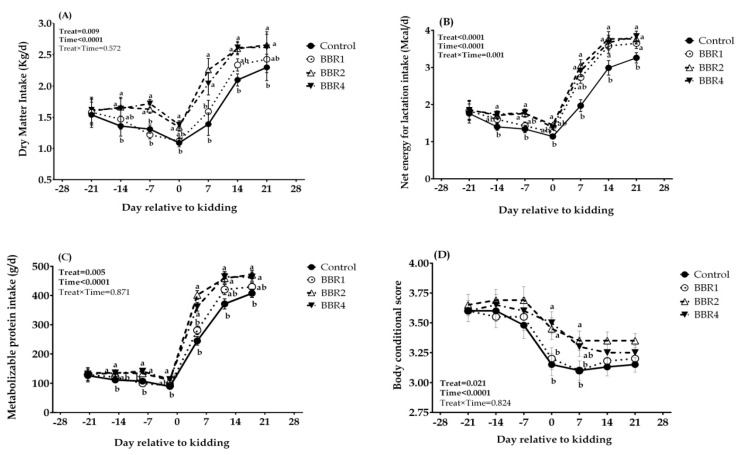
Temporal effects of dietary BBR supplementation on intakes of DM (**A**), NE_L_ (**B**), MP (**C**), and BCS (**D**) in transition dairy Saanen goats from day 21 pre-partum until day 21 post-partum. Data are presented as LSM ± SEM; LSM plots with different lowercase letters (a–b) differ (*p* ≤ 0.05) at the respective time points.

Throughout the pre-partum period, intakes of DM, NE_L_, and MP declined continuously as kidding approached in the CON group, but BBR supplementation increased DM, NE_L_, and MP intakes at both time points, days −14 and −7 before kidding (Figure 1, *p* ≤ 0.05). A sharp decline in DMI that was observed in CON at the kidding day was enhanced with BBR supplementation (*p* ≤ 0.05). Likewise, NE_L_ and MP intakes were higher in BBR-fed goats than the CON group on the kidding day. Over the post-partum period, intakes progressively increased over time in all groups, although supplementing 2 and 4 g/d of BBR resulted an increment in the post-partum DMI at 7, 14, and 21 days after kidding (*p* ≤ 0.05). Overall, BBR increased the intake of DM, NE_L_, and MP (*p* = 0.009, <0.001, and 0.005, respectively), and the overall time effect was observed as the DM, NE_L_, and MP intakes initially decreased pre-partum, followed by a progressive increase in DMI throughout 3 weeks post-partum (time *p* < 0.001). Moreover, the interactions between BBR supplements and time were significant for MP intake (interaction *p* = 0.001).

No differences were observed in pre-partum BCS between the CON and BBR-supplemented groups. At parturition and thereafter, does of the BBR2 and BBR4 groups maintained a better body condition with significantly higher BCS values than CON on days 0 (*p* = 0.045) and 7 (*p* = 0.048). BCS was affected by treatment throughout the transition period (main effect *p* = 0.021), with a noticeable time effect (time *p* < 0.001).

**Figure 2:**
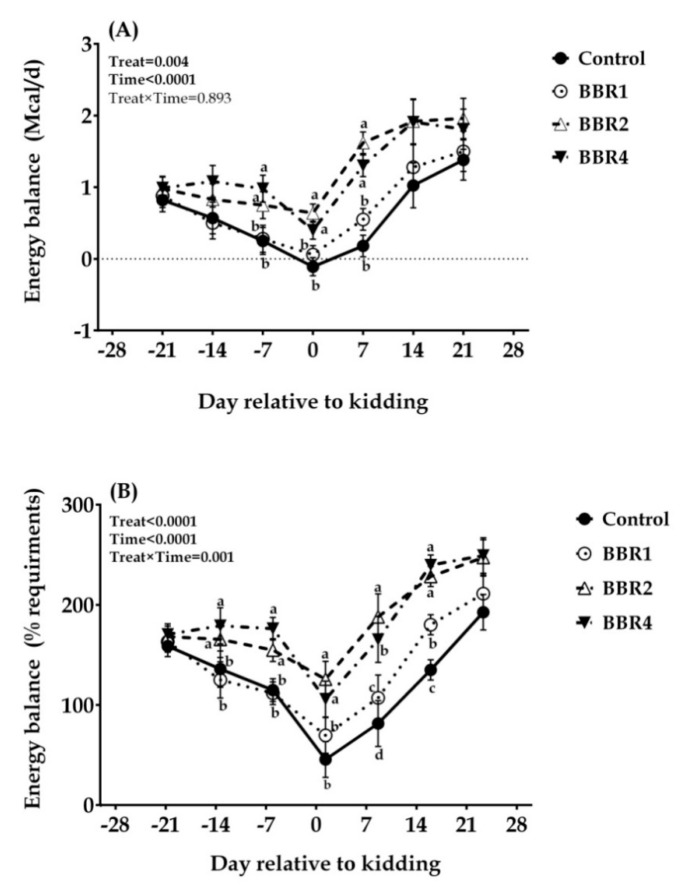
Temporal effects of dietary BBR supplementation on pre-partal and post-partal energy balance as Mcal/d (**A**) or % of requirements (**B**) of transition dairy Saanen goats from day 21 pre-partum until day 21 post-partum. Data are presented as LSM ± SEM; LSM plots with different lowercase letters (a–d) differ (*p* ≤ 0.05) at the respective time points.

With delivery approaching, the energy balance values kept reducing, and NEB appeared in the CON group, whereas the values of BBR-treated goats were still above zero (Figure 2).Both the BBR2 and BBR4 groups elevated the energy balance (as Mcal/d) at days −7, 0, and 7 relative to kidding (*p* = 0.017, 0.046, and <0.001, respectively). Consistently, the energy balance as the percentage of requirements for BBR2 and BBR4 was significantly higher than the CON group whether before or after parturition (except for days−21 and 21). As a whole, the supplemental BBR enhanced the energy balance, both in Mcal/d and in the percentage of requirements (main effect *p* = 0.004 and *p* < 0.001, respectively), and both were altered over time (time *p* < 0.001).

### 3.2. Milk Yield and Milk Components

Both BBR2 and BBR4 elevated the daily milk yield (Table 2; *p* = 0.007). Similarly, the ECM and FCM of BBR-supplemented does were higher than CON (*p* ≤ 0.05), led by BBR2 and followed by BBR4 and BBR1. Of all milk components, only the lactose yield was increased (*p* = 0.04) by BBR supplementation. Additionally, ECM:DMI (*p* = 0.049) and FCM:DMI (*p* = 0.019) were enhanced by supplementation with BBR, as well as milk yield:DMI (*p* = 0.040). The highest values of feed efficiency were observed in BBR2, followed by BBR4 and BBR1.

### 3.3. Biomarkers of Energy Balance and Insulin Resistance

**Figure 3:**
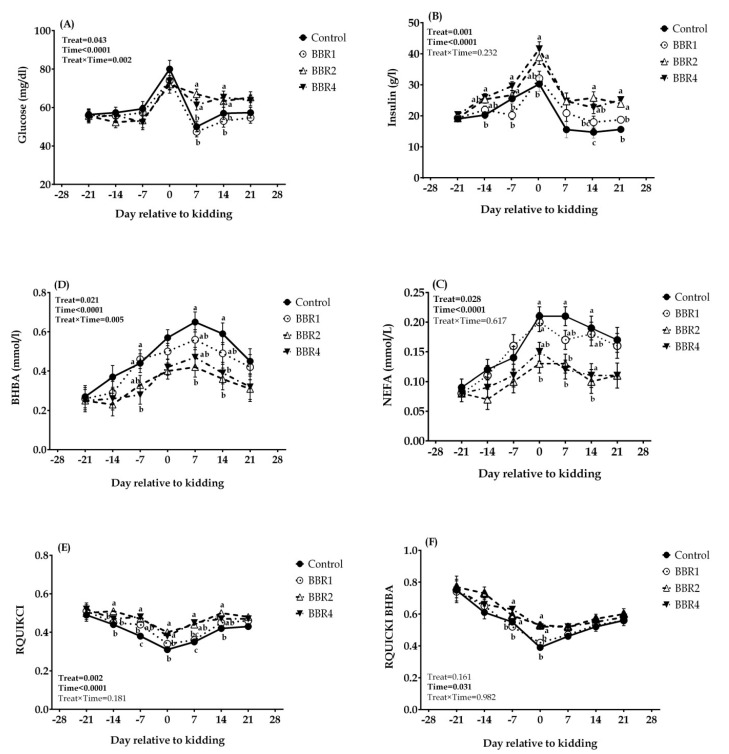
Temporal effects of dietary BBR supplementation on pre-partal and post-partal biomarkers of energy balance and IR: (**A**) Glucose, (**B**) Insulin, (**C**) BHBA, (**D**) NEFA, (**E**) RQUIKCI and, (**F**) RQUIKCI_BHBA_ of transition dairy Saanen goats from day 21 pre-partum until 21 post-partum. Data are presented as LSM ± SEM; LSM plots with different lowercase letters (a–c) differ (*p* ≤ 0.05) at the respective time points.

As shown in Figure 3A, the plasma glucose concentration displayed a sharp elevation on the kidding day in all four groups. During the pre-partum period (days −21, −14, and −7) and at kidding, no significant effect was found among BBR-fed groups and CON. However, on days 7 and 14 after kidding, the glucose concentration in both BBR2 and BBR4 groups was higher than in the CON group (*p* = 0.002 and 0.041, respectively). With increasing BBR supplementation, plasma insulin levels were increased significantly in almost all time points in the transition period (*p* ≤ 0.05), except on day 7 which tended to increase (*p* = 0.10), and on day −21 that was not affected (*p* = 0.767) (Figure 3B).

The weekly plasma NEFA concentration continuously increased over the three weeks after kidding in all groups, which was more pronounced in CON, and then declined progressively from the parturition day until day 21 post-partum (Figure 3C). Supplementing with BBR had no influence on plasma NEFA in the pre-partum period; however, at the parturition day and thereafter, BBR2 and BBR4 reduced NEFA concentrations (*p* ≤ 0.05). The greatest decline in the plasma NEFA concentration was observed in the BBR2 group, followed by the BBR4 group.

Plasma BHBA levels of BBR-fed does were lower than CON, as this decrease had statistical significance on days −7, 7, and 14 relative to parturition (Figure 3D; *p*= 0.040, 0.048, and 0.032, respectively) and tended to decrease at kidding (*p* = 0.10). Furthermore, the BBR2-supplemented goats had the lowest NEFA and BHBA concentrations in most of the transition period. Likewise, insulin resistance was improved with BBR supplementation, as indicated by the higher RQUICKI in BBR2 and BBR4 at days −14, −7, 0, 7, and 14 (Figure 3E; *p* = 0.001, 0.002, 0.003, 0.009, and 0.005, respectively) and RQUICKI_BHBA_ at days −7 and 0 (Figure 3F; *p* = 0.041 and 0.03, respectively). Overall, BBR-treated groups represented elevated glucose and insulin levels as well as reduced NEFA and BHBA (main effect *p* ≤ 0.05). All values were significantly different over the six weeks (time *p* < 0.001). Moreover, the effect of BBR on the plasma glucose and BHBA content showed an interaction with weeks (interaction *p* = 0.002 and *p* = 0.005, respectively).

### 3.4. Biomarkers and Quantification of Liver Function

**Figure 4:**
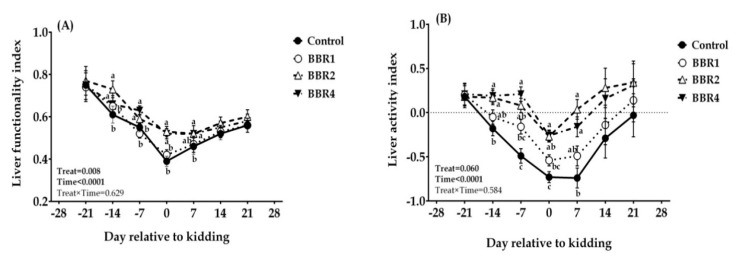
Temporal changes in liver functionality index (LFI, **A**) and liver activity index (LAI, **B**) in transition dairy goats supplemented with different levels of BBR. Data are presented as LSM ± SEM; LSM plots with different lowercase letters (a–c) differ (*p* ≤ 0.05) at the respective time points.

Plasma biomarkers of liver function and capacity in plasma and their quantification are shown in Table 3 and Figure 4, respectively. Plasma activities of AST, ALT, and ALP kept rising during the pre-partum period, followed by a progressive decline after kidding. Neither AST nor ALT significantly changed by BBR supplementation at pre-partum (*p* ≥ 0.05). Nevertheless, at the kidding day and day 7 post-partum, there were lower AST and ALT levels in the BBR2 and BBR4 groups compared with CON (*p* ≤ 0.05). Additionally, the BBR2-fed does showed the lowest concentration of ALP, followed by BBR4 and BBR1 at days −7 and 0 (*p* = 0.001); however, BBR1 was similar to CON. Indeed, BBR-supplemented does showed an enhanced plasma concentration of albumin compared to the CON group, which was led by BBR2 at days −7, 0, 7, 14, and 21 of the transition period (*p* ≤ 0.05).

With parturition approaching, the quantification indices of liver function, LFI, and LAI fell in all treatments, which was associated with the lower severity in BBR-supplemented groups, and then recovered following the occurrence of lactation, with the superiority of BBR-supplemented groups (Figure 4A,B). Hence, supplementation of either BBR2 or BBR4 significantly enhanced the LFI of peri-parturition Saanen does at days −14, −7, 0, 7, and 14 (*p* ≤ 0.05). Similarly, LAI was improved in goats supplemented with BBR, and the highest LAI at peri-partum (days −14 and −7) and the kidding day was observed in the BBR4 group (*p* ≤ 0.05), but it was the highest for BBR2 at day 7 post-partum (*p* ≤ 0.05). Considering the entire transition period, BBR supplementation enhanced the quantification indices of liver function (main effect *p* ≤ 0.05). Both LFI and LAI changed over time (time *p* < 0.001), but no treatment-by-time interaction was observed (*p* ≥ 0.05; Figure 4).

## 4. Discussion

During the transition period, dairy goats undergo dramatic physiological changes due to the NEB and IR, which, coupled with immunosuppression, renders them susceptible to dysfunction associated with an insufficient energy supply [8,39], as with dairy cows [32] and ewes [40]. In this way, the appropriate approaches to alleviating the NEB during the transition period are a top priority in dairy nutrition. The BBR has been shown to have an energy-enhancing effect in humans and rodent animals [30,41,42], but to our knowledge, no investigation has been revealed in dairy goats. In this study, we found that BBR supplementation alleviated the NEB and improved the energy balance as well as insulin resistance, resulting in improved performance and productivity in periparturient Saanen dairy goats.

### 4.1. Dry Matter Intake, Body Condition Score, and Energy Balance

The temporal variations in the DMI in either the CON or BBR-fed groups were in line with previously published data on transition goats [10,43]. Sun et al. [38] revealed that DMI lags behind the sudden energy requirement for milk production and the resultant NEB. The NEB symptoms appear post-partum, but the dynamic changes in the physiological and metabolic status are verified from the pre-partum period [44]. In the current study, BBR2 and BBR4 enhanced pre-partum DMI (at days −14 and −7), where the CON group showed a downward trend. The decreasing DMI as kidding approached in the CON group, confirmed by Zamuner et al. [10], led to a reduction in NE_L_ and MP intakes as well as the NEB. On the contrary, BBR supplementation (2 and 4 g/d) consequently remarkably increased DMI before kidding as well as NE_L_ intake, MP intake, and the energy balance. At kidding, the dramatic decline in DMI, which was observed in CON does, was ameliorated with BBR supplementation. Likewise, the post-partum DMI and energy balance of BBR2- and BBR4-supplemented goats were higher than CON. Although dairy animal studies are not available in regard to BBR, it has been found that BBR (50 and 100 mg/kg BW) improves energy homeostasis in rat models [45]. In addition, supplementation of 100 mg BBR/kg BW significantly increased the feed intake of broilers [46].

Numerous reports [47,48,49] retrieved from both in vivo and ex vivo studies have shown that BBR can improve energy homeostasis by stimulating AMPK activity. Indeed, Yin et al. [50] declared that BBR is an AMP-activated protein kinase (AMPK) activator. AMPK is a key energy-sensing/signaling system in the cells, and activation of AMPK is well known to increase insulin sensitivity and regulate energy homeostasis [51]. Additionally, our findings were confirmed by Sun et al. [38] and Grummer [15], who reported that an increment in DMI results in an enhancement of NE_L_ and MP intakes as well as alleviating the NEB.

Walsh et al. [52] reported that the severe NEB tends to increase fat mobilization, and therefore there are greater losses in BCS at the early post-partum period. This was confirmed by our results that showed either BBR2- or BBR4-supplemented goats had a greater post-partum BCS. The dynamic changes in the EB and BCS in our experiment show that BBR could raise pre- and post-partum DMI, increase the intakes of NE_L_ and MP, ameliorate the lags between DMI and post-partum energy requirements, and thereby alleviate the NEB in transition dairy goats.

### 4.2. Milk Yield and Components

The post-partum milk yield is an important indicator of dairy goat production performance, which is influenced by many factors, such as the pre-parturition energy intake, oxidative stress, and inflammation [53]. Goats undergoing a greater NEB, coupled with increased oxidative stress and inflammation post-partum, are likely to suffer from production inefficiency as well as metabolic disorders [10,53]. The supplementation of BBR did not affect milk fat, total solids, and SNF yield; however, supplementing the diet with 2 and 4 g/d BBR significantly increased the milk yield, and FCM, ECM, and milk lactose yields. The variation in the milk yield is largely explained by the variation in the energy intake and DMI. Our observations of the milk yield, ECM yield, and FCM yield are in agreement with other studies which demonstrated a positive relationship between the EB and milk yield [10,54]. Indeed, Staples et al. [55] indicated that if DMI is not depressed, as a result, the energy balance and milk yield might be improved in early-lactating dairy ruminants [56].

BBR appears to enhance DMI as well as mitigating fat mobilization, with the onset of pregnancy thereupon; the increase in energy intake establishes favorable conditions for milk production. Our observations on feed efficiency are in agreement with other studies [57,58,59] which reported an increase in feed efficiency with an elevation in the energy intake.

### 4.3. Biomarkers of Energy Balance and IR

To adapt to the special physiological state of nutrient deficit in the transition period, goats normally experience an increase in adipose tissue lipolysis due to changes in hormones such as insulin [10,53]; consequently, a large quantity of NEFA are released from adipose tissue into the circulation around kidding or in early lactation and are then transported to the liver where they can be oxidized to provide energy to the liver, partially oxidized to produce ketone bodies, or esterified to TAG [53]. Indeed, a state of insulin resistance may develop as part of the physiologic (pregnancy and lactation) processes, which may manifest as decreased insulin sensitivity [60]. Therefore, NEFA and BHBA are considered effective indicators of the energy status in transition goats [10].

The NEFA suggested values for does, with a neutral EB, are 0.200–0.217 mmol/L [61], which is in line with the present findings. The NEFA had a similar pattern in the CON and BBR groups, which spiked at the kidding day and was in agreement with Zamuner et al. [8]. The decrease in the NEFA concentration caused by either BBR2 or BBR4 supplementation observed in this study is an indicator of ameliorating fat mobilization. To date, the effect of BBR on ruminants has not been studied; however, Li et al. [62] declared that the levels of circulating NEFA decreased by BBR supplementation in male mice, which was related to activating AMPK and improving insulin sensitivity. Importantly, consistent with our results, Shi et al. [63] presented the beneficial effects of BBR on the mitochondrial respiratory chain function and insulin signaling of bovine hepatocytes in a dose-dependent manner.

The BBR-supplemented does represented higher glucose levels immediately after delivery and thereafter, which was mostly associated with a higher DMI and EB. In this regard, a recent paper underlined the fact that the blood glucose level in pregnant dairy sheep was increased with the energy intake [64]. The higher RQUICKI and RQUICKI_BHBA_ in BBR-supplemented goats relative to CON indicate that early-lactating goats fed BBR are less prone to insulin resistance, hence suggesting higher insulin responsiveness, as described by Abuelo et al. [36] in dairy cows. Following our results, it has been demonstrated that BBR activates the AMP-activated protein kinase with beneficial metabolic effects in diabetic and insulin-resistant states [48]. Indeed, it has been approved that BBR could significantly improve the mitochondrial respiratory chain function and insulin signaling damage induced by NEFA through PGC-1 in bovine hepatocytes [63]. Together, these findings indicate the role of BBR in ameliorating IR and enhancing the EB in the transition state. However, activation of AMPK has been linked to insulin sensitivity, although the exact mechanism for this is unknown [65]; hence, the altered insulin sensitivity coupled with the changes in AMPK activation and insulin signaling in BBR-treated goats warrants further investigation.

### 4.4. Biomarkers and Quantification of Liver Function

The activities of AST, ALT, and ALP were discussed together as they are usually used to assess liver function [66]. The reduction in these enzymes in the BBR-treated groups (namely BBR2 and BBR4) implies that the BBR-supplemented does were exposed to a lower degree of liver injury over the transition period, although they had mobilized less fat deposits than CON (as shown by the lower concentrations of NEFA and BHB); the latter finding was confirmed by Abuelo et al. [36] in transition cows. The role of BBR in the treatment of liver dysfunction has been approved in vitro and in vivo [67]. Furthermore, our data are supported by Othman et al. [68], who revealed that the hepatoprotective effects of BBR administration are accompanied by the suppression of AST, ALT, and ALP levels in mercury-induced hepatorenal toxicity in rats. The fact that albumin is one of the indicators of liver function and classified as a negative acute-phase protein implies that its reduction may depress the liver function [14]. Therefore, the substantial increase in albumin in goats that consumed either 2 or 4 g/d BBR immediately before kidding and thereafter compared with CON (Table 3) represents an improved liver function.

Compared with the aforementioned blood biomarkers, the LAI and LFI are more precise for reflecting the liver function during the transition period [37,38]. Although no data are available in the literature on the dynamic changes in the LFI and LAI throughout the transition period of dairy goats, it has been confirmed that transition cows with either a lower LAI or LFI presented a severe NEB, inflammation responses, and liver dysfunction [3,14]. However, Sun et al. [69] reported that BBR is protective against liver dysfunction, as evidenced by decreased ALT and AST activities, as well as being able to mitigate inflammation. Mounting evidence indicates that the underlying mechanisms of BBR in enhancing the liver function might involve the inhibition of oxidative stress, hepatocyte necrosis, and inflammatory responses [70]. Therefore, it can be speculated that BBR was able to enhance the liver functionality of pre-parturition goats by reducing inflammation and mitigating the NEB, consistent with those animals experiencing less pronounced NEB conditions.

## 5. Conclusions

In summary, BBR supplementation (2 and 4 g/d) elevated DMI, energy intake, and milk production as well as feed efficiency in pre- and post-partum goats. The energy balance was improved in goats supplemented with BBR2 and BBR4. Likewise, glucose metabolism and IR parameters were enhanced with BBR treatment, as indicated by the reduction in NEFA and BHBA, and the accession of RQUICKI. Serum hepatic enzyme activities (ALT, AST, and ALP) were suppressed thereupon, in the transition state, whereas albumin and liver activity indicators (LAI and LFI) were enhanced following BBR supplementation. The results suggest that the addition of either 2 or 4 g BBR/d is appropriate to benefit IR and the NEB in pre-parturition goats, which provides a potential new therapeutic strategy for the prevention of metabolic dysfunction in transition dairy goats and promotes an improved lactation performance as well as improved health.

## Figures and Tables

**Table 1:** Ingredients and nutrient composition (DM basis) of pre- and post-partum diets.

Item	Diets	
	Pre-Partum ^a^	Post-Partum ^b^
Ingredient (% of DM)		
Alfalfa hay	4.00	29.5
Corn silage	34.3	10.8
Wheat straw	17.9	5.00
Barley grain, ground	7.70	10.8
Corn grain, ground	31.5	22.2
Soybean meal	1.00	17.0
Wheat bran	1.80	2.20
Calcium carbonate	0.90	1.00
Minerals and vitamins premix ^c^	0.90	0.50
Salt	0.00	1.00
Chemical composition		
ME (Mcal/kg of DM)	2.60	2.90
NE_L_ (Mcal/kg of DM)	1.60	1.82
CP (% DM)	18.5	15.5
Ether extract (% DM)	2.50	2.50
Ash (% DM)	7.60	8.00
NDF (% DM)	43.0	37.3
NFC (% DM) ^d^	38.0	36.7

^a^ From 50 days before parturition until kidding. ^b^ From kidding until 21 days of lactation. ^c^ Containing vitamin A (250,000 IU/kg), vitamin D (50,000 IU/kg), vitamin E (1500 IU/kg), manganese (2.25 g/kg), calcium (120 g/kg), zinc (7.7 g/kg), phosphorus (20 g/kg), magnesium (20.5 g/kg), sodium (186 g/kg), iron (1.25 g/kg), sulfur (3 g/kg), copper (1.25 g/kg), cobalt (14 mg/kg), iodine (56 mg/kg), and selenium (10 mg/kg). ^d^ Non-fibrous carbohydrates (NFC) were estimated according to the equation: NFC = 100 − (NDF + CP + EE + Ash) [31].

**Table 2:** Lactation performance, milk composition, and feed efficiency of BBR-supplemented Saanen dairy goats throughout the transition period.

Item ^1^	Treat ^2^				SEM	*p*-Values
	Control	BBR1	BBR2	BBR4		
Post-partum DMI, kg/d	2.21 ^b^	2.28 ^b^	2.47 ^a^	2.42 ^a^	0.058	0.021
Milk yield, kg/d	1.66 ^b^	1.96 ^b^	2.48 ^a^	2.10 ^ab^	0.143	0.007
ECM, kg/d	1.69 ^b^	2.15 ^a^	2.77 ^a^	2.21 ^ab^	0.131	0.002
4% FCM, kg/d	1.62 ^b^	2.12 ^ab^	2.68 ^a^	2.11 ^ab^	0.152	0.003
Milk Fat						
Yield, kg/d	0.08	0.09	0.11	0.09	0.012	0.219
Content, %	4.68	4.74	4.55	4.11	0.582	0.697
Milk protein						
Yield, kg/d	0.07	0.08	0.09	0.08	0.008	0.451
Content, %	4.56	3.92	3.76	3.72	0.363	0.314
Milk lactose						
Yield, kg/d	0.06 ^b^	0.09 ^ab^	0.11 ^a^	0.10 ^ab^	0.012	0.042
Content, %	3.94	4.47	4.52	4.70	0.252	0.224
Milk total solids						
Yield, kg/d	0.23	0.28	0.31	0.27	0.028	0.287
Content, %	14.02	13.89	12.16	12.86	0.596	0.144
Milk total solids non-fat						
Yield, kg/d	0.15	0.19	0.22	0.20	0.018	0.147
Content, %	9.28	9.27	8.61	9.31	0.196	0.078
Feed efficiency						
Milk yield: DMI, kg/d	0.80 ^b^	0.94 ^ab^	1.09 ^a^	0.99 ^a^	0.061	0.050
ECM: DMI, kg/d	0.97 ^b^	1.17 ^ab^	1.31 ^a^	1.13 ^ab^	0.085	0.050
FCM: DMI, kg/d	0.82 ^b^	1.01 ^ab^	1.18 ^a^	1.00 ^ab^	0.070	0.019

^a,b^ Means with different superscript letters within a row are different (*p* ≤ 0.05). ^1^ Post-partum data cover the first 21 days of lactation. ^2^ Control, BBR1, BBR2, and BBR4 supplemented with 0, 1, 2, and 4 g/d BBR, respectively.

**Table 3:** Plasma biomarkers of liver functionality in transition dairy Saanen goats supplemented with different levels of BBR.

Item	Time ^1^	Treat ^2^				SEM	*p*-Values
		Control	BBR1	BBR2	BBR4		
AST (U/L)	−21	73.50	75.34	75.60	73.67	4.173	0.839
	−14	74.16	78.30	74.66	69.00	4.320	0.532
	−7	86.16	83.66	73.34	71.00	5.590	0.225
	0	93.17 ^a^	87.70 ^ab^	70.34 ^b^	70.84 ^b^	5.625	0.044
	7	94.15 ^a^	87.00 ^a^	75.84 ^b^	79.50 ^ab^	3.804	0.037
	14	93.17	90.00	78.17	80.83	4.281	0.107
	21	89.50	85.34	77.50	75.50	5.525	0.307
ALT (U/L)	−21	23.00	22.00	22.67	23.8	1.35	0.837
	−14	24.00	22.34	23.00	19.00	1.715	0.265
	−7	25.34	24.33	20.65	18.67	1.83	0.101
	0	29.33 ^a^	25.00 ^ab^	19.00 ^b^	20.65 ^b^	1.433	0.003
	7	29.00 ^a^	23.67 ^ab^	19.66 ^b^	20.66 ^ab^	2.041	0.045
	14	28.33	23.60	21.66	22.00	1.802	0.101
	21	28.00	23.00	20.34	21.00	2.944	0.316
ALP (U/L)	−21	129.00	135.66	131.33	131.00	11.904	0.881
	−14	141.67	140.33	131.00	127.00	11.880	0.783
	−7	168.00 ^a^	153.33 ^ab^	122.34 ^c^	134.33 ^bc^	5.300	0.001
	0	201.67 ^a^	176.66 ^ab^	146.00 ^c^	151.67 ^bc^	6.258	0.001
	7	178.00 ^a^	160.34 ^ab^	137.00 ^bc^	125.00 ^c^	6.888	0.026
	14	173.00	160.33	136.67	141.00	15.91	0.116
	21	171.67	170.00	129.00	142.00	17.91	0.287
Albumin (g/dL)	−21	4.47	4.73	4.60	4.46	0.230	0.820
	−14	4.47	4.43	4.90	5.03	0.182	0.111
	−7	4.33 ^c^	4.47 ^bc^	5.23 ^a^	4.97 ^ab^	0.151	0.010
	0	3.73 ^c^	3.80 ^bc^	4.36 ^ab^	4.50 ^a^	0.140	0.009
	7	3.87 ^c^	4.13 ^bc^	5.03 ^a^	4.67 ^ab^	0.171	0.005
	14	3.97 ^b^	3.86 ^b^	5.24 ^a^	4.74 ^a^	0.212	0.005
	21	3.50 ^c^	4.13 ^bc^	5.26 ^a^	4.96 ^ab^	0.200	0.001

^a,b,c^ Means with different superscript letters within a row are different (*p* ≤ 0.05). ^1^ Day relative to kidding. ^2^ Control, BBR1, BBR2, and BBR4 supplemented with 0, 1, 2, and 4 g BBR/d, respectively.

## Data Availability

The data presented in this study are available on request from the corresponding author (N.G.).
